# Identification of Potential Bioactive Compounds in Strong-Flavor-Type Baijiu via Integration of Widely Targeted Metabolomics and Network Pharmacology

**DOI:** 10.3390/foods15091509

**Published:** 2026-04-27

**Authors:** Jinxiao Liu, Jia Zheng, Jihong Wu, Ying Sun, Mingquan Huang, Jian Su, Fuping Zheng, Dongrui Zhao

**Affiliations:** 1 Key Laboratory of Brewing Molecular Engineering of China Light Industry, Beijing Technology and Business University, Beijing 100048, China; liujx1218986@163.com (J.L.); btbusunying@163.com (Y.S.); huangmq@th.btbu.edu.cn (M.H.); zhengfp@btbu.edu.cn (F.Z.); zdr@btbu.edu.cn (D.Z.); 2 Solid-State Fermentation Resource Utilization Key Laboratory of Sichuan Province, Wuliangye Yibin Co., Ltd., Yibin 644000, China; zhengjia@wuliangye.com.cn (J.Z.); sujian@wuliangye.com.cn (J.S.); 3 Key Laboratory of Wuliangye-Flavor Liquor Solid-State Fermentation, China National Light Industry, Wuliangye Yibin Co., Ltd., Yibin 644000, China; 4 China Engineering Technology Research Center for Baijiu-Making Grains, Wuliangye Yibin Co., Ltd., Yibin 644000, China

**Keywords:** bioactive components, network pharmacology, strong-flavor-type Baijiu, widely targeted metabolomics

## Abstract

Strong-flavor-type Baijiu, represented by *Wuliangye*—a renowned traditional Chinese alcoholic beverage brewed from five grains (sorghum, rice, glutinous rice, wheat, and corn)—is widely consumed and appreciated for its balanced taste and potential health benefits. While the volatile flavor compounds of Baijiu have been well studied, its bioactive components and their underlying mechanisms remain insufficiently explored. In this study, widely targeted metabolomics techniques were innovatively employed, and 2128 compounds were identified from 10 *Wuliangye* samples, of which 445 were predicted to constitute potential bioactive substances. Network pharmacology analysis further identified four key compounds, namely the four potential bioactive small molecules (fisetin, luteolin, norartocarpetin, and scutellarein), along with ten core targets that were key protein targets interacting with these compounds (SRC, PIK3R1, PTGS1, AKR1B1, STAT3, CYP3A4, ESR1, PIK3CA, PIK3CB, and ALOX15). GO and KEGG enrichment analyses indicated that these targets participated in diverse biological processes, while DO analysis revealed potential associations between these targets and specific diseases. Additionally, molecular docking confirmed the binding patterns between the identified compounds and their targets. Collectively, this study provides systematic chemical information and theoretical screening results for identifying potential bioactive components in strong-flavor-type Baijiu, which may facilitate further studies of their biological functions.

## 1. Introduction

Baijiu, a national alcoholic beverage of China with a history spanning over 2000 years, is a typical grain-based distilled spirit. It is produced through a unique solid-state brewing process, including raw material crushing, microbial saccharification by qu, long-term solid-state fermentation in cellars, distillation, aging storage, and scientific blending [1,2]. This traditional and elaborate brewing procedure promotes the formation of abundant flavor substances and diverse bioactive components, thereby providing a material foundation for its quality and potential health benefits [3]. Advanced and widely used analytical methods have been widely applied to characterize the aroma components of Baijiu [4,5], with over 2700 compounds identified, including volatile phenols, esters, and aldehydes, as well as non-volatile amino acids, polyols, and bioactive peptides [6]. Among the various types of Baijiu, the strong-flavor-type Baijiu is globally renowned for its distinctive brewing process and superior quality, and a total of 861 trace components (e.g., 248 esters, 122 alcohols, and 24 phenols) have been reported in this type [7].

Notably, the quality of Baijiu is determined not only by its volatile flavor substances but also by non-volatile trace components with potential health benefits. With increasing health awareness, research on healthy Baijiu has become a focus. Moderate consumption of Baijiu has been reported to benefit health, which is likely attributed to its bioactive components [7,8]. For example, *Wuliangye* Baijiu, as a representative of strong-flavor-type Baijiu, was shown to promote intestinal homeostasis in mice by regulating intestinal stem cell proliferation, differentiation and gut microbiota composition [9], while pyrazines in sauce-flavor-type Baijiu exhibited anti-inflammatory effects via modulation of the NF-κB/MAPK pathway [10]. However, the molecular mechanisms underlying the bioactivity of these compounds remain insufficiently analyzed. Thus, identifying the functional components in Baijiu and their health benefits is critical for advancing the industry’s high-quality development.

In recent years, metabolomics has emerged as a powerful analytical tool for conducting qualitative and quantitative analyses of numerous metabolites in biological samples, thereby offering novel approaches to investigating the functional components within complex systems [11]. Meanwhile, network pharmacology integrates research methods from multiple disciplines, including systems biology, medicinal chemistry, molecular biology, bioinformatics, and computational chemistry. By constructing networks between drugs and biological targets, this approach facilitates a deeper understanding of the mechanisms of action and the multi-target effects of drugs, thereby providing strong support for elucidating the mechanisms of action of functional components [12,13]. Despite their potential, these methods have rarely been applied in Baijiu research.

Prior studies had confirmed that Baijiu and its by-products were rich in bioactive substances, including polyphenols, terpenoids, bioactive peptides, and polysaccharides, which exhibited antioxidant, anti-inflammatory, blood pressure-lowering, and intestinal microbiota-regulating activities [14]. However, obvious limitations remain in the research. Specifically, most studies focus primarily on a single type of bioactive component (e.g., peptides, polysaccharides, or phenolics) and lack systematic profiling of the overall metabolome in Baijiu. Furthermore, the research approaches are predominantly centered on compound identification and in vitro activity validation, with rare involvement of target prediction, signaling pathways, and underlying molecular mechanisms. In particular, comprehensive analysis and mechanistic research of non-volatile bioactive components in strong-flavor Baijiu are still insufficient. For instance, Zeng et al. focused on the preparation, purification, identification, and functional validation of bioactive peptides from Baijiu and its fermentation byproducts, yet comprehensive profiling of the global metabolome was not performed [15]. Hu et al. merely conducted widely targeted metabolomics profiling and in vitro evaluations of the antioxidant and antiglycation activities of Baijiu distillers’ grains without exploring potential targets or regulatory pathways [16]. Similarly, Guan et al. only reviewed the extraction and bioactivities of functional components from Baijiu distillers’ grains without in-depth mechanistic investigation [17]. In particular, comprehensive profiling and mechanistic studies of non-volatile bioactive components in Baijiu are still inadequate. Therefore, it is urgent to establish a systematic strategy to globally reveal the bioactive components and their potential health regulatory mechanisms in Baijiu.

Therefore, to address the aforementioned critical limitations, this study integrated widely targeted metabolomics and network pharmacology to identify and analyze bioactive components in *Wuliangye* Baijiu samples. Initially, ultra-high-performance liquid chromatography–electrospray–triple-quadrupole linear ion trap tandem mass spectrometry (UPLC-ESI-Q TRAP-MS/MS) was used for comprehensive metabolite profiling of the *Wuliangye* samples. Subsequently, potential functional components, their targets, and bioactivities were predicted through network pharmacology, followed by compound–target–disease network construction to explore mechanisms. This study overcame the limitations of previous research that only focused on the activity analysis of single components. Furthermore, it addressed the research gap regarding the insufficient mechanistic exploration of non-volatile bioactive components in strong-flavor Baijiu. Consequently, the systematic identification and analysis of functional components in strong-flavor Baijiu and their potential health benefits are of significant importance. These findings will help to scientifically reveal the health attributes of Baijiu, standardize the healthy development of the industry, and meet consumers’ growing demand for healthy alcoholic beverages. Thus, exploring the functional components in Baijiu and their associated health benefits is crucial for advancing the industry’s high-quality development. Notably, ethanol is the main component of Baijiu. Excessive or long-term consumption of Baijiu may be associated with potential adverse health effects. This study focuses solely on the identification and screening of trace components and their potential physiological activities in Baijiu, which does not reflect the overall effects of Baijiu itself.

## 2. Materials and Methods

### 2.1. Wuliangye Baijiu Sample Treatment

The primary objective of this study was to characterize the global profile of bioactive components in *Wuliangye* Baijiu collectively rather than to compare differences among individual products. Thus, a total of 10 distinct *Wuliangye* Baijiu products were procured for this study from Yonghui Supermarket (Beijing, China) and the official *Wuliangye* flagship store on JD.com (Table 1). These samples were then divided into two groups based on product characteristics and research focus. One group consisted of a single sample, WU LIANG YE (Eighth Generation, Yibin, China), which was selected separately due to its distinctive flavor, high market popularity, broad consumer recognition, and superior quality compared to other variants. For this group, 50 mL of Baijiu was subjected to atmospheric distillation at a heating temperature of 90 °C, and the final volume of the sample was reduced to 20 mL. The other group included the remaining nine *Wuliangye* Baijiu products. An amount of 35 mL of each of these 9 products was measured out and then mixed in equal proportions to form a composite sample. This composite sample was then subjected to atmospheric distillation under the same conditions (heating temperature of 90 °C). The final combined volume of the composite sample was 140 mL, corresponding to a concentration factor of 2.25, the same as for the first group. To ensure the repeatability and reliability of the results, three technical replicates were analyzed for each sample during instrumental analysis.

### 2.2. Widely Targeted Metabolomics Analysis and Compounds Database Building

**Figure 1:**
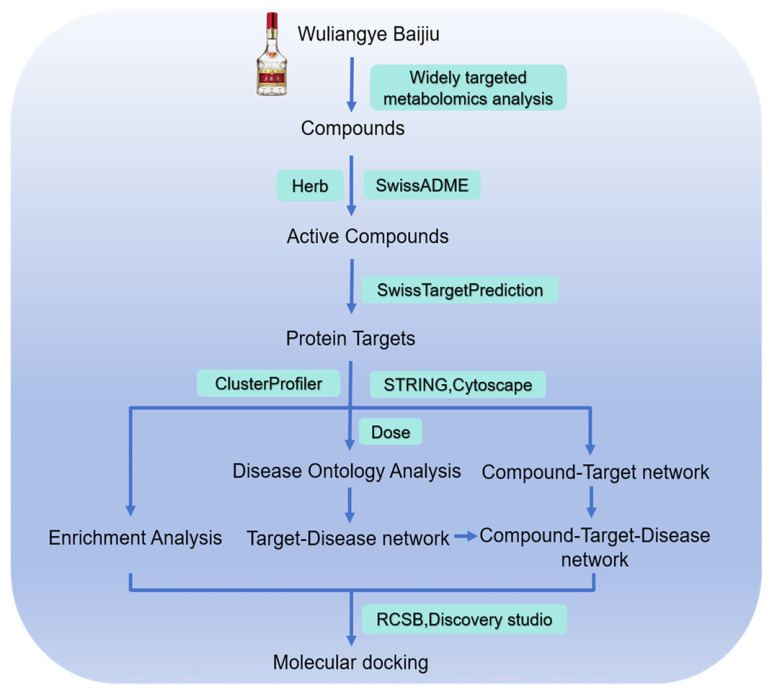
Diagram of the methodology applied in this study.

The workflow of this study is presented in Figure 1. The sample preparation and extraction were conducted as previously described, with minor modifications [18]. Briefly, the treated Baijiu samples were freeze-dried and then added to a 70% methanol internal standard extract. The internal standard used was L-2-chlorophenylalanine at a concentration of 1 µg/mL. After lyophilization, the samples were redissolved in the same extractant and concentrated 30-fold for signal correction and quantitative analysis. Notably, this internal standard was not employed for absolute quantitation of metabolites but rather to monitor the stability of the instrumental system throughout the widely targeted metabolomics analysis. The mixture was stirred for 15 min and centrifuged at 12,000 r/min at 4 °C for 3 min. The supernatant was filtered through a microporous membrane (0.22 μm) and then analyzed using a UPLC-ESI-Q TRAP-MS/MS system (UPLC: Nexera X2, Shimadzu, Kyoto, Japan; MS: 6500 Q TRAP, SCIEX, Framingham, MA, USA). Quality control (QC) samples were prepared by mixing equal aliquots of all sample extracts. During instrumental analysis, one QC sample was inserted every 10 injections to monitor system stability and analytical repeatability. For UPLC analysis, an Agilent SB-C18 column (1.8 µm, 2.1 mm × 100 mm) was used. The mobile phase consisted of solvent A (water with 0.1% formic acid) and solvent B (acetonitrile with 0.1% formic acid). The gradient elution program started at 95% A and 5% B, changed to 5% A and 95% B within 9 min, held for 1 min, and then returned to 95% A and 5% B within 1.1 min and held for 2.9 min. The flow rate was maintained at 0.35 mL/min, the column temperature was set to 40 °C, and the injection volume was 2.0 µL. For ESI, the source temperature was 500 °C, the ion spray voltage was 5500 V (positive mode) or −4500 V (negative mode), and gases were set at GSI: 344.74 kPa, GSII: 413.69 kPa, and CUR: 172.37 kPa. The instrument was tuned and calibrated with polypropylene glycol solutions (10.0 and 100 µmol/L) in QQQ and LIT modes. QQQ scans were performed in MRM mode with optimized DP and CE for each transition. The Metware database was used to match parameters, including retention time (RT), secondary mass spectrometry fragment ions, parent ion molecular weight, characteristic fragment ion, DP, and CE of the substances. Metabolites were quantified in multiple reaction monitoring (MRM) mode using triple-quadrupole mass spectrometry.

Metabolite annotation was performed via a widely targeted metabolomics approach based on UPLC-ESI-Q TRAP-MS/MS and multiple reaction monitoring (MRM) scanning, referencing to the Metware database. A strict three-level qualitative identification system was applied: Level 1 (full matching of MS/MS fragment ions and RT, score ≥ 0.7), Level 2 (full matching of MS/MS fragment ions and RT, score 0.5–0.7), and Level 3 (consistent matching of Q1, Q3, RT, DP, and CE parameters). The identification level for each metabolite is listed in Table S1.

### 2.3. Screening Potential Active Compounds and ADMET Evaluation

Traditional Chinese medicine provides a rich foundation for modern drug discovery and development. The Herb database (http://herb.ac.cn) is a specialized high-throughput experimental and reference database for traditional Chinese medicine [19]. ADMET (absorption, distribution, metabolism, excretion, and toxicity) is a key evaluation parameter in druglikeness studies. In this study, the web tool SwissADME (http://www.swissadme.ch/ (accessed on 26 April 2025)) was used to evaluate the ADMET parameters. SwissADME provided information regarding various chemical properties of small molecules, including ADME parameters, physicochemistry, druglikeness, pharmacokinetics, and medicinal chemistry friendliness properties [20]. The potential active compounds were screened based on the following criteria [21]: (1) the compound components must be retrievable from the Herb database, (2) a minimum of two “YES” results for druglikeness properties (Lipinski, Ghose, Veber, Egan, Muegge), (3) the gastrointestinal absorption (GI) level must be “high”.

### 2.4. Target Prediction and Interaction Network Construction

The SwissTargetPrediction (http://www.swisstargetprediction.ch/) is a powerful online tool which predicts potential protein targets by comparing the two-dimensional (2D) and three-dimensional (3D) structural similarities between the query molecule and known ligands [22]. Here, SwissTargetPrediction was used to predict the targets of each potential active compound. The SMILES identifiers of the filtered compounds were imported into SwissTargetPrediction with the “Homo sapiens” setting. A higher probability of a protein target generally correlates with greater accuracy of the prediction. Therefore, a probability threshold of 0.4 was set to filter out more credible protein targets and enable a reliable compound–target (C-T) network. Given that protein–protein interaction (PPI) networks can complement and improve a C-T network, a PPI network was constructed using STRING (https://string-db.org/). The filtered protein targets were imported into STRING with “Homo sapiens” as the “organism” and the “minimum required interaction score” set to “highest confidence (0.900)” [23].

### 2.5. Topological Analysis of Compound–Target Network

The network was visualized using Cytoscape v.3.10.3 [24]. To identify the key active compounds and core targets, CytoHubba was employed. CytoHubba can comprehensively evaluate the key nodes in the network through various topological analysis methods [23,25]. The top 10 core nodes of the C-T network were analyzed using 10 different methods, including edge percolated component, maximum neighborhood component, density of maximum neighborhood component, maximal clique centrality, and six centralities (degree, closeness, betweenness, bottleneck, eccentricity, and stress). Subsequently, the final core components were determined by selecting nodes that were consistently identified as significant by at least three of the topological analysis methods [26].

### 2.6. GO and KEGG Enrichment Analyses

To explore the biological effects of the filtered protein targets, the R package clusterProfiler was used to conduct GO enrichment analysis and KEGG pathway analysis [27]. The *p*-value was adjusted using the Benjamini–Hochberg method, and results with a *p*-value < 0.05 were considered significant. Then, ClueGO and CluePedia, two Cytoscape plugins, were employed to further visualize and interpret the enrichment results. The relationships between pathways and the enrichment of genes within those pathways were more intuitively displayed by integrating ClueGO and CluePedia [28]. For this analysis, the filtered target gene symbols were input into the “Functional analysis” mode, and the “Homo sapiens (9606)” option was selected. The p-value was corrected using a two-sided hypergeometric test and the Bonferroni step down method.

### 2.7. Disease Ontology Analysis

The Human Disease Ontology (DO; https://www.disease-ontology.org) database is a comprehensive knowledge base that contains a variety of human diseases, thereby providing standardized disease classification and descriptions [29]. DO enrichment analysis is a method employed to identify the gene sets related to specific diseases. Potential biological significance and disease associations can be revealed through DO analysis. Here, the DO analysis was performed using the R package DOSE [30]. For the analysis, the Benjamini–Hochberg method was used to adjust the p-value, with significance set at a *p*-value of less than 0.05.

### 2.8. Compound–Target–Disease Network Construction

Based on the establishment of the C-T network and DO analysis, the complex relationships between active compounds, protein targets, and diseases were visualized using Cytoscape. In the compound–target–disease (C-T-D) network, nodes represent active compounds, protein targets, or diseases, while edges represent the interactions among them. The importance of compounds and targets was assessed; the higher the degree, the greater its importance.

### 2.9. Molecular Docking Analysis

To validate the reliability of the C-T association, molecular docking analysis was performed using the filtered active compounds and targets. Briefly, the docking process was carried out as follows: (1) the 3D structure of the compound (ligand) in SDF format was downloaded from the PubChem database, (2) the crystal structure of the protein target (receptor) in PDB format was downloaded from the RCSB website, (3) the original co-crystallized ligand and water molecules from the receptor were removed using discovery Studio 2019 (DS) software; then, molecular docking was performed with the ligand. For each ligand, ten binding poses were generated. The CDOCKER energy (based on the CHARMm force field) was used as the scoring function to evaluate the relative binding affinity between ligands and the target.

### 2.10. Data Processing

Pie charts were constructed using GraphPad, and network diagrams were generated using Cytoscape. The GO and KEGG databases were utilized for the annotation of metabolic pathways.

## 3. Results and Discussion

### 3.1. Identification of the Metabolites of Wuliangye Baijiu

Based on the mass-to-charge ratio in the Metware database, a total of 2128 metabolites were identified in *Wuliangye* Baijiu (as detailed in Table S1) for the first time, including 281 terpenoids, 276 alkaloids, 234 lipids, 212 amino acids and derivatives, 210 flavonoids, 196 phenolic acids, 112 organic acids, 69 lignans and coumarins, 50 nucleotides and derivatives, 30 quinones, six tannins, five steroids, and 447 unclassified compounds. The comprehensive effect of these compounds might contribute to both the flavor and health effects of Baijiu, as further analyzed below.

Terpenoids constituted the largest proportion among the ten kinds of *Wuliangye* Baijiu samples, which was likely due to the unique brewing process and raw material selection, as reported by Feng et al., that systematically tracked and analyzed terpenoid compounds throughout the brewing process of strong-aroma Baijiu. That process involved raw materials, daqu (a type of fermentation starter), cellar mud, wine dregs, and base Baijiu [31]. Meanwhile, a total of 55 terpenoids were identified in Moutai Baijiu—a typical representative of soy-sauce-flavor-type Baijiu, which were regarded to provide more elegant and delicate aroma to Moutai Baijiu [32]. Therefore, these terpenoids might not only endow *Wuliangye* Baijiu with a unique fragrance but may also offer health benefits such as antibacterial [33], antioxidant [34], anticancer [35], and stomach protection [36]. Alkaloids—a class of basic nitrogen—constituted the second-most abundant group after terpenoids in *Wuliangye* Baijiu. Most alkaloids exhibited significant physiological activity, making them effective components in many medicinal plants. For example, narciclasine had shown significant mitosis-blocking activity in various cancer cell lines [37]. Betaine exerts anti-inflammatory effects in numerous diseases by improving sulfur amino acid metabolism to combat oxidative stress, inhibiting NF-κB signaling and NLRP3 inflammasome activation, regulating energy metabolism, and alleviating endoplasmic reticulum stress and apoptosis [38]. Dopamine can modulate immune cells in the brain and periphery, while immune cells can also indirectly affect distant tissues through dopaminergic regulation [39]. Free fatty acids are the main lipids in *Wuliangye* Baijiu. These compounds are not only precursors for the formation of the main aromatic fatty acids esters in the liquor but also play an important role in the formation of the aroma and aftertaste of the Baijiu [40]. Myristoleic acid can inhibit the activation of Src and Pyk2 and block the rearrangement of the cytoskeleton and the formation of actin rings. As a result, it can suppress the maturation of osteoclasts and bone resorption, exhibiting potential therapeutic value in the prevention of osteoporosis and other bone metabolic diseases [41]. Punicic acid exerts positive effects against neurodegenerative diseases by reducing oxidative damage and inflammation through the upregulation of peroxisome proliferator-activated receptors. Additionally, it decreases the formation of beta-amyloid deposits and tau hyperphosphorylation by increasing the expression of GLUT4 protein and inhibiting calpain hyperactivation [42]. Similarly, other polyunsaturated fatty acids have been shown to exert positive effects on cardiovascular diseases [43], the nervous system [44], and overall immunity [45].

In addition, amino acids and derivatives, flavonoids, and phenolic acids collectively accounted for a significant proportion (29.1%) of the substances detected in *Wuliangye* Baijiu. Proteins in the raw materials were broken down by microorganisms into amino acids during the brewing process of Baijiu, which were subsequently converted into flavor-specific esters and other compounds during fermentation, thereby enhancing the aromatic complexity of the Baijiu [46]. Amino acids themselves possessed umami, sweet, bitter, astringent, and sour taste properties, thereby directly influencing the mouthfeel and flavor profile [47]. Flavonoids, representing 10% of the compounds in *Wuliangye* Baijiu, are bioactive substances with notable antioxidant [48], antibacterial [49], and antiviral properties [50]; they can react with other compounds in the Baijiu to form new flavor substances [51]. Phenolic compounds contributed aromas, such as smoky, muddy and fruity, to Baijiu [52,53], playing critical roles in its aroma, taste, and stability. Phenolic acids like guaiacol derivatives acted as natural free radical scavengers with potent reactive oxygen species (ROS) elimination capabilities, offering antioxidant effects and potential cardiovascular disease prevention [54].

In contrast, organic acids, lignans and coumarins, nucleotides and derivatives, quinones, tannins, and steroids were present in relatively low concentrations in *Wuliangye* Baijiu. Despite their relatively low content, these compounds might still play significant roles in the overall flavor, taste, and potential health benefits of Baijiu. Organic acids provide a refreshing sour taste and interact with alcohols and esters in the Baijiu to enhance the complexity and layering of the taste [55]. Quinones exhibit redox properties and participate in the oxidation reactions of the Baijiu, influencing its color and stability [56]. Lignans and coumarins, nucleotides and derivatives, tannins, and steroids were all reported to possess antioxidant and antibacterial bioactivities, but further research is required to explore their specific roles and underlying mechanism in Baijiu.

Collectively, these results indicated that the active compounds in *Wuliangye* Baijiu possessed a range of potential biological activities. It should be noted that these conclusions were based on prior studies and in silico analysis, with no experimental validation conducted in the present study. The potential effects of these compounds after ingestion, digestion, and absorption in vivo warrant further investigation.

### 3.2. Prediction of Potential Active Compounds and Their Pharmacological Properties

**Figure 2:**
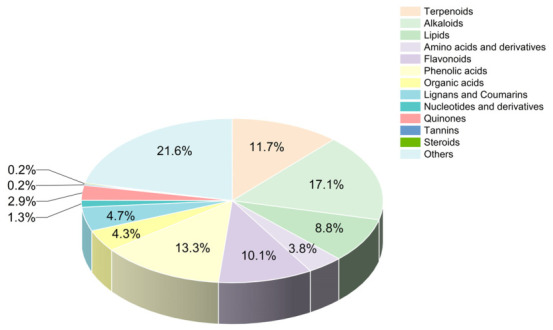
Pie chart of the number of different types of potential active compounds in *Wuliangye* Baijiu.

After screening the Herb database and analyzing the data with SwissADME, a total of 445 potential active compounds were identified based on the screening criteria outlined in Section 2.3 (listed in Table S2). Alkaloids, phenolic acids, terpenoids, and flavonoids accounted for 52.2% of the total active components and were the main active compounds in *Wuliangye* Baijiu. The relative proportions of these potential active compounds within *Wuliangye* Baijiu were visually represented in Figure 2. The analysis revealed that the average molecular weight of these potential active compounds was approximately 218.13, with a range from 88.15 to 678.81. According to the predicted pharmacological properties, 65.62% (292/445) of the active compounds exhibited high water solubility, 67.87% (302/445) could penetrate the blood–brain barrier, 88.31% (393/445) were not substrates of P-glycoprotein, and 35.06% (156/445) of the active compounds fully complied with Lipinski’s rule of five, indicating their high druglikeness.

### 3.3. Compound–Target Interaction Network

To gain further insight into the mechanisms of these potential active compounds in the human body, the possible protein targets were predicted. Targets with a probability of ≥0.4 were selected to construct a C-T network (Table S3). The results revealed that 207 targets were associated with 127 active compounds. To further explore the key components in *Wuliangye* Baijiu, topological analysis was performed using multiple methods, with the results presented in Table 2. Furthermore, the common components identified by at least three topological methods are listed in Table S4. In total, four key active compounds, including fisetin, luteolin, norartocarpetin, and scutellarein, together with ten core targets (SRC, PIK3R1, PTGS1, AKR1B1, STAT3, CYP3A4, ESR1, PIK3CA, PIK3CB, and ALOX15), were obtained, which was an important new discovery in this study. In the C-T network, fisetin, luteolin, norartocarpetin, and scutellarein were connected to multiple target proteins. Similarly, the ten core target proteins were linked to various active compounds. Therefore, these compounds and targets were recognized as key contributors to the active effects of *Wuliangye* Baijiu.

In addition, it was noteworthy that luteolin, norartocarpetin, and scutellarein belong to the flavonoid class of compounds. Fisetin, a natural flavonoid polyphenolic compound widely distributed in fruits, vegetables, and natural plants, had been shown to promote the activation of Caspase-3 to induce apoptosis by regulating the PI3K/ AKT/NF-κB axis [57]. It can mediate the TLR4/Src pathway to inhibit the expression of the NF-κB p65 and MAPK signaling pathways, thus alleviating lipopolysaccharide-induced renal inflammation in mice and reducing the expression of inflammatory factors [58]. Moreover, fisetin treatment had also been reported to alleviate cognitive deficits in aged SAMP8 mice, while restoring their impaired synaptic function and stress response, indicating that it had certain antiaging effects [59]. Luteolin, another natural flavonoid compound, also exhibited a wide range of health benefits. Specifically, it alleviated CCl4-induced liver inflammation and fibrosis in mice by inhibiting macrophage infiltration and modulating M1 macrophage polarization via the Notch1 signaling pathway [60]. Additionally, luteolin inhibited the production of proinflammatory mediators (IL-1β, IL-6, IL-8, IL-17, IL-22, TNF-α, and COX-2) and regulated various signaling pathways (NF-κB, JAK-STAT, and TLR signaling pathways) to modulate numerous inflammatory processes in the skin [61]. Norartocarpetin, a compound in Artocarpus communis that demonstrates antioxidant and antityrosinase activity, downregulated the expression of phospho-cAMP response element-binding (phospho-CREB) and microphthalmia-associated transcription factor (MITF), which reduced the synthesis of tyrosinase and intracellular melanin content, thereby inhibiting melanogenesis [62]. Scutellarin exerted antitumor effects by inducing autophagy and apoptosis in tumor cells, as well as inhibiting proliferation, migration, and invasion of tumor cells [63]. Additionally, scutellarin was an effective GABA-T (Gamma-Aminobutyric Acid Transaminase) inhibitor, with an IC50 value of (12.8 ± 1.0) mmol/L, and its inhibitory effect on SSADH (succinic semialdehyde dehydrogenase) was more obvious, with an IC50 value of only (7.20 ± 0.9) mmol/L, which revealed its neuroprotective potential in ischemic brain injury, epilepsy, and other diseases [64]. Collectively, these findings suggest that the four flavonoid compounds might exhibit a range of physiological activities as functional components in *Wuliangye* Baijiu. However, their actual activities in Baijiu and the related mechanisms were not experimentally verified in this study. It should also be noted that flavonoid concentrations in distilled spirits are generally low. Therefore, their actual concentrations, physiological relevance, and biological activities after oral administration and alcohol metabolism will require further verification in subsequent studies.

### 3.4. Enrichment Analysis of the Core Protein Targets

**Figure 3:**
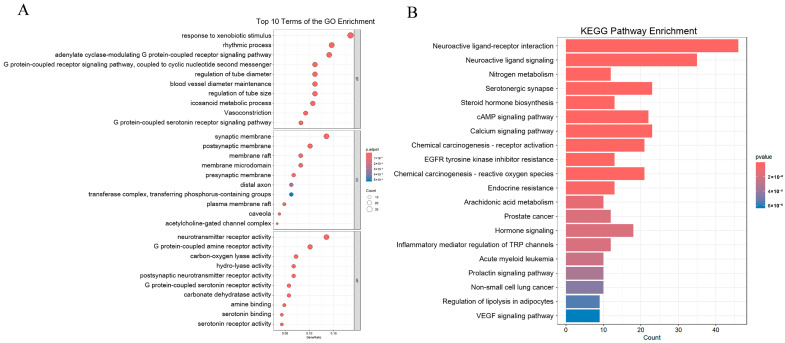
(**A**) Bar chart of GO enrichment analysis (top 10 in every category) of the targets (p.adjust < 0.05); (**B**) dotplot of top 20 signal pathways based on KEGG enrichment analysis (p.adjust < 0.05).

**Figure 4:**
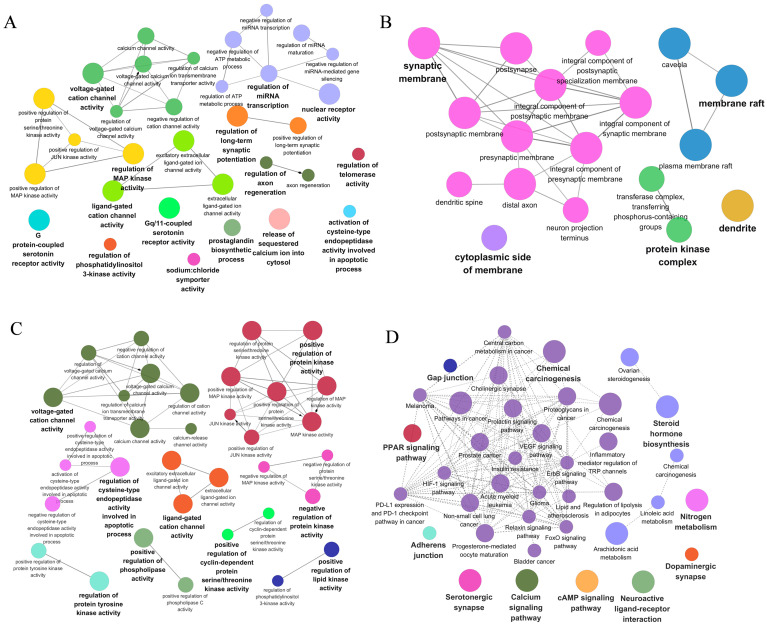
(**A**) Enrichment analysis of protein targets in biological process; (**B**) enrichment analysis of protein targets in cellular component; (**C**) enrichment analysis of protein targets in molecular function; (**D**) enrichment analysis of protein targets in metabolic pathways. Nodes represent significantly enriched GO terms, with size proportional to the number of enriched target genes (larger nodes = more genes). Node colors distinguish distinct functional clusters of related GO terms. Edges indicate shared target genes. All GO terms shown in the network were screened with an adjusted *p*-value < 0.05 to ensure statistical significance.

To explore the biological functions of the protein targets, 207 protein targets were imported into clusterProfiler for GO and KEGG enrichment analysis. The detailed results are respectively listed in Tables S5 and S6. A total of 1376 biological processes, 57 molecular functions, 190 cellular components, and 255 signaling pathways were obtained. The top ten significantly enriched GO terms and the top twenty significantly enriched KEGG pathways were visualized in Figure 3A,B, respectively. To further analyze the interactions between these protein targets and their functions in different pathways, ClueGO and CluePedia were used to cluster GO and KEGG terms with similar functions together. The terms were then merged and filtered to select the more representative ones. The relationships between gene functions were more intuitively displayed using network visualization, and the results are shown in Figure 4A–D. From this analysis, the conclusion could be easily obtained that for BP, the potential biological effects of the impact were related to regulation of miRNA transcription, nuclear receptor activity, voltage-gated cation channel activity, regulation of long-term synaptic potentiation, regulation of MAP kinase activity, ligand-gated cation channel activity, regulation of axon regeneration, G protein-coupled serotonin receptor activity, regulation of phosphatidylinositol 3-kinase activity, Gq/11-coupled serotonin receptor activity, sodium: chloride symporter activity, the prostaglandin biosynthetic process, the release of sequestered calcium ion into cytosol, activation of cysteine-type endopeptidase activity involved in the apoptotic process, and regulation of telomerase activity (Figure 4A). For CC, the potential biological effects of the impact were related to the synaptic membrane, cytoplasmic side of the membrane, protein kinase complex, dendrite, and membrane raft (Figure 4B). For MF, the potential biological effects of the impact were related to voltage-gated cation channel activity, negative regulation of protein kinase activity, regulation of cysteine-type endopeptidase activity involved in the apoptotic process, positive regulation of phospholipase activity, ligand-gated cation channel activity, positive regulation of lipid kinase activity, positive regulation of cyclin-dependent protein serine/threonine kinase activity, positive regulation of protein kinase activity, and regulation of protein tyrosine kinase activity (Figure 4C). In addition, the highly enriched signal pathways were mainly involved in the PPAR signaling pathway, gap junction, adherens junction, serotonergic synapse, calcium signaling pathway, cAMP signaling pathway, neuroactive ligand–receptor interaction, dopaminergic synapse, steroid hormone biosynthesis, nitrogen metabolism, and chemical carcinogenesis (Figure 4D). These results may be related to the potential biological functions of *Wuliangye* Baijiu.

Moreover, the enrichment analysis also indicated that the functional components in *Wuliangye* Baijiu might broadly affect cellular physiological functions through various mechanisms involving cell signal transduction and regulation, gene expression and transcriptional control, neurophysiological functions, and cellular metabolism and apoptosis, as well as inflammation and immune responses. These functional components significantly influenced cellular response to external stimuli through the modulation of key signaling pathways, including the PPAR signaling pathway, calcium signaling pathway, and cAMP signaling pathway. These signaling pathways played crucial roles in fundamental processes, including cell proliferation, differentiation, metabolism, and apoptosis. For instance, PPARγ promoted lipid synthesis and fat storage by inducing the expression of adipocyte-specific genes such as FAS in adipose tissue, which helped regulate fatty acid metabolism and energy storage to maintain the homeostasis of adipose tissue [65]. Regulation of the calcium signaling pathway activates the influx of calcium ions into cells. Inhibition of calcium ion channels in satellite cells has been shown to significantly impact cellular activation and proliferation [66]. Additionally, functional components also regulated the activity of voltage-gated and ligand-gated ion channels, affecting the ion balance inside and outside of the cell. This regulation subsequently influenced neural signal conduction [67] and muscle contraction [68], both of which are vital for maintaining normal cellular physiological functions. With respect to neurophysiological function, these functional components significantly influenced neural signal transduction by modulating neuroactive ligand–receptor interactions, serotonin receptor activity, and dopaminergic synaptic function. These modulatory effects are of great importance for maintaining the normal function of the nervous system [69]. In cellular metabolism and apoptosis, functional components regulate the activity of PI3K, phospholipase, and lipase, which influences metabolic processes within the cell. These modulatory effects impact not only cellular energy metabolism [70] but also cell survival and apoptosis [71].

### 3.5. Disease Ontology Analysis

**Figure 5:**
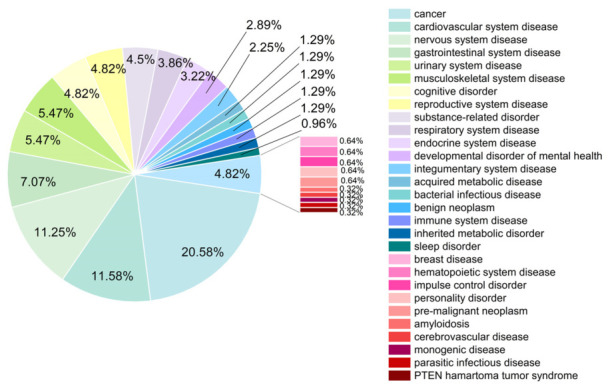
Pie plot of disease ontology terms of 207 targets. Percentages were calculated based on 311 disease entries and rounded for clarity. The total sum may not equal 100% due to rounding.

DO analysis enabled the identification of genes associated with specific diseases and facilitated the construction of disease-related gene or protein networks. In this study, the set of 207 protein targets was imported into DOSE for DO enrichment analysis to explore the diseases they may affect. The results showed that the 207 protein targets were associated with 311 diseases (Table S7). To more systematically understand the relationships between these diseases and their hierarchical positions within disease classification, the diseases were further categorized into primary and secondary entries, as detailed in Table S7. The 311 diseases were classified into 29 secondary entries and seven primary entries (Figure 5). Of the 207 protein targets, 57.8% targets were related to anatomical entity disease, 22.5% to cellular proliferation disease, 14.4% to mental health disease, 2.8% to metabolism disease, 1.6% to infectious agent disease, 0.3% to genetic disease, and 0.3% to syndromes. In the secondary disease categories, the top four diseases were cancer, cardiovascular system disease, nervous system disease, and gastrointestinal system disease, which collectively accounted for 50.48% of the target-associated diseases. Taken all together, these computational predictions suggest that the active compounds in *Wuliangye* Baijiu may be potentially associated with regulating the development of cancer, cardiovascular system disease, nervous system disease, and gastrointestinal system disease by modulating protein targets. It should be emphasized that these were theoretical inferences from network pharmacology and thus require future experimental validation.

### 3.6. Compound–Target–Disease Network Analysis

**Figure 6:**
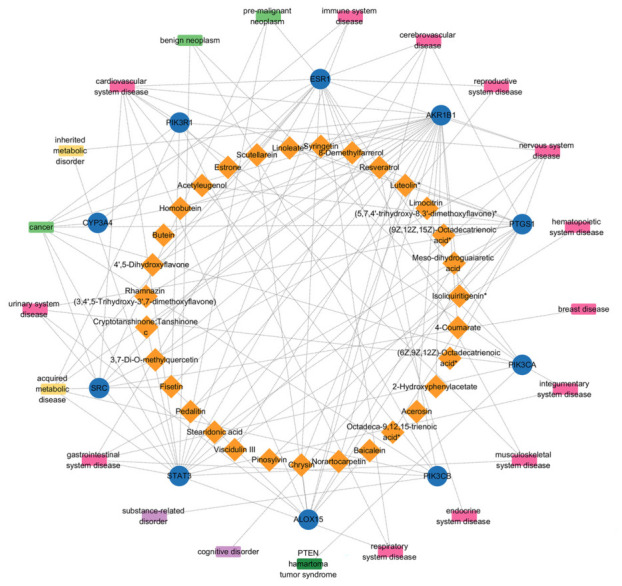
Compound–target–disease (C-T-D) networks of 10 core targets of *Wuliangye* Baijiu. Orange diamond nodes represent key active compounds, blue circular nodes represent core target proteins, and colored rectangular nodes represent related disease categories. Edges denote interactions or associations between nodes: compound–target edges indicate predicted binding, and target–disease edges indicate biological associations. * indicates that the corresponding compound has indistinguishable stereoisomers.

Based on the C-T network obtained in Section 3.3 and the gene–disease relationships obtained in Section 3.5, a C-T-D network (Figure 6) was successfully constructed using the ten core targets (SRC, PIK3R1, PTGS1, AKR1B1, STAT3, CYP3A4, ESR1, PIK3CA, PIK3CB, and ALOX15) as connecting nodes. The resulting network comprised 62 nodes and 117 edges. In addition to the four key compounds, the ten core targets were also potential targets for multiple compounds. In primary disease classification, the diseases associated with the targets were anatomical entity disease, metabolism disease, cellular proliferation disease, mental health disease, and syndromes. In secondary disease classification, SRC was associated with the regulation of seven diseases, PIK3R1 with six diseases, PTGS1 with seven diseases, AKR1B1 with five diseases, STAT3 with 16 diseases, CYP3A4 with two diseases, ESR1 with 12 diseases, PIK3CA with seven diseases, PIK3CB with six diseases, and ALOX15 with four diseases. Notably, STAT3 and ESR1 were associated with the greatest number of disease types. STAT3 was a transcriptional regulatory factor with DNA-binding activity which regulated various biological processes, including cell proliferation, differentiation, autophagy, apoptosis, and inflammation. It regulated the growth hormone of lysine via the JAK2-STAT pathway, thereby promoting the transcription and expression of genes involved in cell growth and metabolism in adipose tissue, liver, muscle, and bone [72]. Additionally, STAT3 has been shown to regulate Ca^2+^ flux by downregulating endoplasmic reticulum IP3R3, preventing the excessive release of proapoptotic factors, and modulating the activity of Bcl-2 family proteins to exert antiapoptotic functions [73]. Furthermore, STAT3 also regulated T cell responses to external stimuli, including proinflammatory cytokines and chemokines [74]. ESR1 is a member of the steroid hormone nuclear receptor family of transcription factors and exhibits broad functions. Specifically, it can inhibit lipid formation and white fat deposition by binding to target genes or regulating the transcription factors of key genes in adipocyte differentiation to reduce their phosphorylation [75]. ESR1 also regulates the expression of related genes, including Col1a1, Col1a2, and Col3a1, to protect the liver from fibrosis [76]. Collectively, the interactions of these protein targets and their compounds may contribute to the potential regulatory effects of *Wuliangye* Baijiu.

### 3.7. Molecular Docking Verification

Elucidating the molecular recognition mechanisms and associated intermolecular interactions through simulation of the spontaneous binding process of biomolecules was crucial for understanding compound–target interaction [77]. The interactions between the receptor and ligand typically included hydrogen bonds, electrostatic interactions, van der Waals forces, and hydrophobic interactions. The CDOCKER energy score was employed as a relative scoring metric to assess the potential binding affinity between small-molecule ligands and the target protein. It should be clearly stated that the CDOCKER energy score represents a relative empirical metric for ranking the binding affinity of ligands to the target protein. This score does not represent the true thermodynamic ΔG (Gibbs free energy) value. A more negative CDOCKER energy value generally indicates a stronger predicted binding capacity of the ligand to the target [78]. Based on the four key active compounds and the ten core targets, a total of 45 compound–target docking pairs were identified. The docking results of the computational docking simulation study are shown in Table S9. To assess the reliability of the docking protocol, redocking validation of the co-crystallized ligands was performed for six of the ten core targets (excluding PIK3R1, STAT3, CYP3A4, and PIK3CB). The root mean square deviation (RMSD) values of these six validated targets were all below 2.0 Å, confirming the reliability of the docking results for these targets. For the remaining four targets (PIK3CB, PIK3R1, STAT3, and CYP3A4), high-quality, drug-like co-crystallized ligands suitable for redocking validation could not be identified in the PDB database (available structures only contained cofactors, small-molecule additives, or modified residues), so redocking validation could not be performed. Therefore, the docking results of these four targets should be considered as predictive and require further experimental verification in the future.

**Figure 7:**
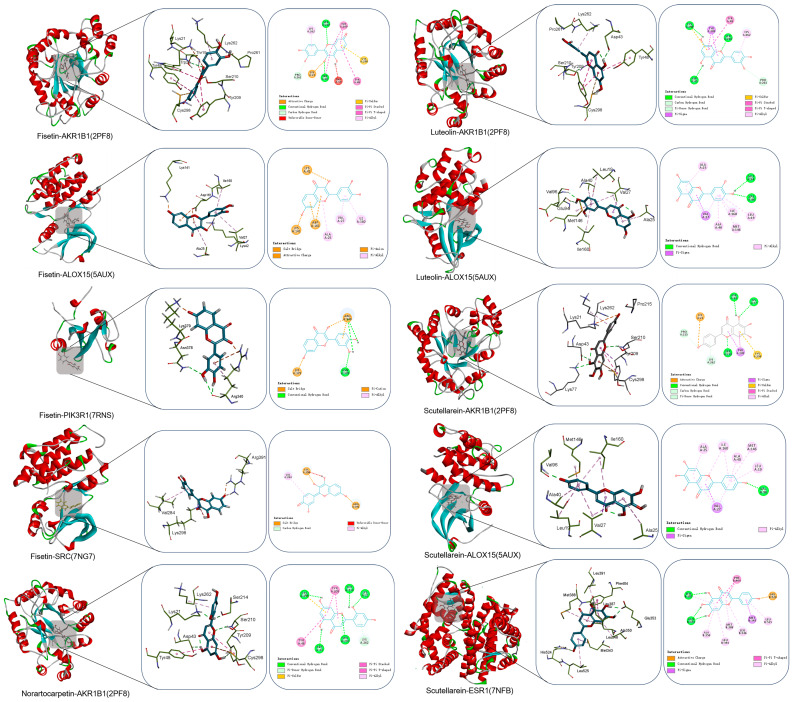
Molecular binding pattern of the ten complexes.

The docking results showed that all the CDOCKER energy values were less than 0 kcal/mol^−1^, except for the docking of stearidonic acid with PTGS1, indicating that the ten core targets exhibited good docking characteristics and binding affinity with their active compounds. Notably, the CDOCKER energy values of the complexes formed between the key compounds and their respective core targets were all lower than −30 kcal/mol^−1^, indicating high binding affinities and stability between them. The binding affinities of the ten compounds–targets were attributed to hydrogen bonds, hydrophobic interactions, and van der Waals forces from multiple amino acid residues (Figure 7). Taking Fisetin-ALOX25 as an example, fisetin interacted with Lys141, Asp161, Ala25, and Val27 through hydrogen bonds and contacts with amino acid residues Ile160, Lys52, Lys141 through hydrophobic interactions. These interactions collectively promoted the tight binding between the compounds and their targets.

Overall, the molecular docking results indicated that the majority of target–compound pairs exhibited favorable binding characteristics, with the exception of the interaction between stearidonic acid and PTGS1. In particular, the complexes formed between the key compounds and their core targets exhibited tight association through various types of interactions. Notably, the docking results of the six validated targets (with RMSD < 2.0 Å) were reliable, while the results of the four targets without redocking validation were predictive and needed further experimental confirmation. These findings were consistent with the results of the preceding network pharmacology analyses, further supporting the potential of these compounds as functional components in *Wuliangye* Baijiu.

## 4. Conclusions

This study innovatively integrated widely targeted metabolomics and network pharmacology approaches, establishing a novel systematic analytical method for identifying functional components in Baijiu. Through this approach, four key active compounds, including fisetin, luteolin, norartocarpetin, and scutellarein, were identified for the first time. Then, network pharmacology revealed their potential interactions with multiple biological targets. Moreover, ten core targets, including SRC, PIK3R1, PTGS1, AKR1B1, STAT3, CYP3A4, ESR1, PIK3CA, PIK3CB, and ALOX15, were ultimately identified. These targets played critical roles in diverse physiological and pathological processes involving inflammation, oxidative stress, cell signaling, immune response, and other aspects. Both network analysis and subsequent molecular docking validation revealed complex interactions between the key active compounds and their targets, which may theoretically contribute to the potential health-related properties of *Wuliangye* Baijiu.

However, it should be acknowledged that the present research is mainly based on in vitro analysis and computational simulation, lacking the separation and extraction of these bioactive compounds, as well as more comprehensive in vivo studies. Therefore, further research is warranted to elucidate the specific efficacy and potential mechanisms of these compounds.

## Figures and Tables

**Table 1:** Different types of *Wuliangye* Baijiu.

Group	Name	Alcohol Content (*v*/*v* %)	Production Date
1	WU LIANG YE (Eighth Generation)	52	2024/02/05
2	WU LIANG CHUN	52	2024/03/11
	WU LIANG CHEN	52	2021/07/13
	WU LIANG TOU QU	52	2023/09/27
	WU LIANG CHUN (premium)	52	2024/01/08
	WU LIANG TE QU	52	2022/08/30
	FU GUI TIAN XIA JIU	52	2023/12/28
	ZUNJIU	52	2022/03/23
	WU LIANG CHEN (Heritage Series)	52	2021/04/26
	JIAN ZHUANG	52	2024/01/23

**Table 2:** Top 10 core compositions of the compound–target (C-T) network.

Betweenness	Bottleneck	Closeness	Degree	DMNC	Eccentricity	EPC	MCC	MNC	Stress	Rank
Fisetin	Fisetin	Fisetin	Fisetin	EGFR	CA13	Fisetin	SRC	SRC	PTGS1	1
PTGS1	Scutellarein	Norartocarpetin	Norartocarpetin	HSD17B3	CA5A	Luteolin	PIK3R1	Fisetin	Fisetin	2
Nuciferine	ALOX5	Luteolin	Luteolin	IGF1R	AKT1	SRC	EGFR	ESR1	ALOX15	3
DRD4	DRD4	Scutellarein	SRC	PTK2	CA9	PIK3R1	PTK2	AKR1C3	Luteolin	4
Luteolin	PTGS1	CA2	STAT3	CYP2C9	CA14	Norartocarpetin	IGF1R	PIK3R1	CYP3A4	5
MAOA	AKR1B1	AKR1B1	CYP3A4	PIK3R1	CA6	STAT3	PIK3CA	PIK3CA	(9Z,12Z,15Z)—octadecatrienoic acid	6
Norartocarpetin	(9Z,12Z,15Z)—octadecatrienoic acid	SRC	Scutellarein	Nicotine	CA3	PIK3CA	PIK3CB	CYP3A4	FABP4	7
Resveratrol	Nuciferine	CA12	ESR1	CHRNA7	SRC	ESR1	CYP2C19	STAT3	FABP3	8
AKR1B1	CA2	MAOA	AKR1C3	CHRNA3	CA1	Scutellarein	CYP2C9	ALOX15	ALOX5	9
SLC6A4	ACHE	CA7	PIK3R1	CHRNB4	ADORA2A	PIK3CB	ALOX15	PIK3CB	Linoleate	10

## Data Availability

The original contributions presented in this study are included in the article and Supplementary Materials. Further inquiries can be directed to the corresponding author.

## Supplementary Materials

The following supporting information can be downloaded at: https://www.mdpi.com/article/10.3390/foods15091509/s1, Table S1: a list of 2128 compounds from *Wuliangye* Baijiu; Table S2: the physicochemical properties, pharmacokinetics parameters, and druglikeness of 445 potential active compounds; Table S3: a list of the interactions between 127 active compounds and 207 targets; Table S4: the shared compositions of the targets and the active compounds in *Wuliangye* Baijiu; Table S5: the list of biological processes (BPs), molecular function (MF), and cellular components (CCs) category terms from GO enrichment analysis; Table S6: the list of key pathway terms from KEGG enrichment analysis; Table S7: the list of significant DO terms and their parent terms; Table S8: The list of the compound–target–disease network based on 10 key targets; Table S9: The parameter and result of molecular docking.

## Abbreviations

The following abbreviations are used in this manuscript:

NF-κB	nuclear factor-κB
PI3K	phosphatidylinositol-3 kinase
AKT	protein kinase B
GO	Gene ontology
KEGG	Kyoto Encyclopedia of Genes and Genomes
DO	Disease ontology
MAPK	mitogen-activated protein kinase
SRC	Tyrosine-protein kinase SRC
PIK3R1	PI3-kinase p85-alpha subunit
PTGS1	Cyclooxygenase-1
AKR1B1	Aldose reductase
STAT3	Signal transducer and activator of transcription 3
CYP3A4	Cytochrome P450 3A4
ESR1	Estrogen receptor alpha
PIK3CA	PI3-kinase p110-alpha subunit
PIK3CB	PI3-kinase p110-beta subunit
ALOX15	Arachidonate 15-lipoxygenase
